# Medical Image Classifications for 6G IoT-Enabled Smart Health Systems

**DOI:** 10.3390/diagnostics13050834

**Published:** 2023-02-22

**Authors:** Mohamed Abd Elaziz, Abdelghani Dahou, Alhassan Mabrouk, Rehab Ali Ibrahim, Ahmad O. Aseeri

**Affiliations:** 1 Department of Mathematics, Faculty of Science, Zagazig University, Zagazig 44519, Egypt; 2 Artificial Intelligence Research Center (AIRC), Ajman University, Ajman 346, United Arab Emirates; 3 Faculty of Computer Science & Engineering, Galala University, Suze 435611, Egypt; 4 Department of Electrical and Computer Engineering, Lebanese American University, Byblos P.O. Box 36, Lebanon; 5 Mathematics and Computer Science Department, University of Ahmed DRAIA, Adrar 01000, Algeria; 6 Mathematics and Computer Science Department, Faculty of Science, Beni-Suef University, Beni-Suef 62521, Egypt; 7 Department of Computer Science, College of Computer Engineering and Sciences, Prince Sattam Bin Abdulaziz University, Al-Kharj 11942, Saudi Arabia

**Keywords:** Internet of medical things, 6G networks, deep learning, feature selection, arithmetic optimization algorithm, hunger games search, metaheuristic

## Abstract

As day-to-day-generated data become massive in the 6G-enabled Internet of medical things (IoMT), the process of medical diagnosis becomes critical in the healthcare system. This paper presents a framework incorporated into the 6G-enabled IoMT to improve prediction accuracy and provide a real-time medical diagnosis. The proposed framework integrates deep learning and optimization techniques to render accurate and precise results. The medical computed tomography images are preprocessed and fed into an efficient neural network designed for learning image representations and converting each image to a feature vector. The extracted features from each image are then learned using a MobileNetV3 architecture. Furthermore, we enhanced the performance of the arithmetic optimization algorithm (AOA) based on the hunger games search (HGS). In the developed method, named AOAHG, the operators of the HGS are applied to enhance the AOA’s exploitation ability while allocating the feasible region. The developed AOAG selects the most relevant features and ensures the overall model classification improvement. To assess the validity of our framework, we conducted evaluation experiments on four datasets, including ISIC-2016 and PH2 for skin cancer detection, white blood cell (WBC) detection, and optical coherence tomography (OCT) classification, using different evaluation metrics. The framework showed remarkable performance compared to currently existing methods in the literature. In addition, the developed AOAHG provided results better than other FS approaches according to the obtained accuracy, precision, recall, and F1-score as performance measures. For example, AOAHG had 87.30%, 96.40%, 88.60%, and 99.69% for the ISIC, PH2, WBC, and OCT datasets, respectively.

## 1. Introduction

The emergence of the Internet of medical things (IoMT) and 6G technologies has provided the medical field with a new opportunity and methodologies to improve the diagnosis and prediction of diseases [1,2,3,4]. A large quantity of data, including computed tomographic (CT) images, are generated quickly at a short timescale, raising the problem of efficiently processing such images in real-time to help the medical field detect cancerous diseases in their early stages [5,6,7]. However, the availability and accessibility of medical images have always been limited for researchers due to privacy concerns, holding back the desired rapid advancement in the healthcare domain. Meanwhile, CT images are of low resolution, noisy, and difficult to process, which challenges routine diagnosis in terms of accuracy and precision [8,9,10,11].

In the era of advanced communication technologies such as 6G, providing a real-time medical diagnosis is a critical issue [12]. An early detection of diseases affecting sensitive human body areas, such as blood, breast, lung, and skin, can help limit the spread of the disease and protect the affected body parts. Without providing an accurate and quick diagnosis, the spread of diseases and tumors can be enormously infectious, which can cause a high rate of mortality [13]. For instance, skin cancer detection and prediction is a significant challenge in medical imaging, which is still under development. The health sector can benefit from the rapid development of medical equipment, communication technologies, and the IoMT to provide quality service at an efficient scale.

The IoMT is a collection of internet-related devices that assist healthcare procedures and activities [14]. The IoMT relates to employing the intelligent Internet of things (IoT) and modern communications to service medical personnel, medicines, and medical equipment and facilities to enable the gathering, monitoring, controlling, and a faster access to personal health data. IoT techniques’ software in medicine covers nearly all field areas, including physician ID, remote hospital emergency services, home healthcare of medical products and replacement parts, hospital instruments and the clinical waste surveillance of medical devices, blood management staff, infectious disease control, and others. In addition, the 6G-enabled IoMT provides ultrafast and accurate responses while reducing the workload and the cost of the research and development of the medical field. It is anticipated that the 6G communications system will play an essential role in providing the necessary transmission rate, stability, accessibility, and architecture [15]. Compared to the traditional diagnosis methods used for cancerous disease detection at an early stage, the 6G-enabled IoMT provides a necessary platform for processing enormous healthcare data, including hundreds of slices of CT scans [16,17].

Although the 6G-enabled IoMT has proven helpful for developing embedded systems that can detect illness with the same precision as an expert, it relies heavily on the developed algorithms based on deep learning (DL) and optimization algorithms [18]. The embedded systems in the 6G-enabled IoT can benefit from the capability of DL methods in medical image processing and cancerous disease identification. The adoption of DL methods may assist in avoiding recurrent problems that require a considerable time to solve and the need for a large number of well-labelled training data. Therefore, transfer learning (TL) can assist in overcoming some of these issues by integrating pretrained DL models [19]. DL models incorporate several techniques, such as structure design, model training, model size, feature representation, and hyperparameter optimization. On the other hand, metaheuristic (MH) optimization techniques have proven effective in addressing various complicated optimization issues for computer-aided diagnosis. For instance, Silva et al. [20] improved the hyperparameters of a CNN using particle swarm optimization (PSO) to reduce the false positives (FPs), while detecting lung nodules in lung scans owing to their similar patterns and low population density, which can produce misleading data. Moreover, Surbhi et al. [21] utilized adaptable PSO to automatically diagnose brain tumors to reduce noise and enhance image quality.

This paper introduces a framework to improve the diagnostic imaging identification efficiency, designed to be integrated into the 6G-enabled IoMT. In addition, it aims to overcome several problems, including (1) the curse of dimensionality, (2) the slow inference, and (3) the low performance. The framework comprises two phases: (1) a feature extraction using a deep learning model with transfer learning and (2) a feature selection using a developed optimization algorithm. In the first phase, a developed deep learning architecture is constructed based on MobileNetV3, which acts as a core component for feature extraction. The pretrained MobileNetV3 is employed to learn and extract medical image representations during the deep learning model training using CT images. The pretrained MobileNetV3 has been selected as the deep-learning-based model of choice due to its lightweight design that can be operated on resource-constrained devices with limited energy and resource consumption. In the second phase, a newly developed feature selection method is introduced to enhance the behaviour of the arithmetic optimization algorithm using hunger games algorithms, named AOAHG. The AOAHG method selects only the most relevant features and ensures the overall model classification improvement and efficiency. A thorough assessment of the suggested framework is presented and compared to various state-of-the-art methods utilizing four real-world datasets. In general, the main motivation for using this combination between the AOA, HGS, and MobileNetV3 was based on the shown performance of each performance on different applications. HGS has been applied to handle engineering problems [22], crisis events detection [23], node clustering and multihops [24], feature selection [25], and others [26]. AOA has been applied to solve structural design optimization [27], functionally graded material [28], robot path planning [29], human activity recognition [30], and others [31]. Therefore, this combination can improve the performance of medical image classifications in a 6G IoT-enabled smart health environment. To the best our knowledge, this is the first time the HGS, AOA, and MobileNetV3 have been integrated under a single framework for IoMT.

The main contributions of this work can be summarized as follows:

1. Proposing a 6G-enabled IoMT method that reduces human involvement in medical facilities while providing rapid diagnostic results. The new method is designed to be integrated into resource-constrained systems.
2. Using the transfer learning approach to extract the features from the medical images.
3. Enhancing the ability of the arithmetic optimization algorithm as a feature selection technique using operators of the hunger games search.
4. Evaluating the developed 6G-IoMT model using four datasets and comparing its performance with other state-of-the-art techniques.

The rest of this paper provides a background on transfer learning for extracting features. Section 4 provides the developed 6G-enabled IoMT framework. Section 5 discusses the image diagnosis framework’s outcomes. Lastly, in Section 6, the conclusion and future scope are discussed.

## 2. Related Works

The power of classification to aid in medical diagnosis makes it an important field of research. As a result of classification optimization, researchers have improved classification performance by applying deep learning and transfer learning in the IoMT. In addition, using metaheuristic optimization algorithms in conjunction with convolution neural networks (CNN) for medical image classification is presented in this section. Table 1 summarizes the literature review on the used datasets in our study.

### 2.1. IoMT-Based Deep Learning

Due to the spread of contagious diseases which can cause a pandemic, a reliable infrastructure offering conventional diagnostic tools and systems has emerged in the IoMT. The IoMT relies on the IoT infrastructure, which lowers the information transmission latency and the complexity of centralized diagnosis processes. The IoMT offers several solutions for the medical field, including monitoring systems, medical information-sharing mediums, remote consulting, and automatic report generation. Thus, technology facilitates the life of patients by offering to monitor and consult systems while helping the medical staff by reducing human intervention and human faults. The IoMT collects information from the patient using different types of sensors, devices, and clinical records, which can be stored and shared on a cloud-based centre [32]. For instance, computed aided diagnosis (CAD) technologies rely on IoT technologies to offer medical image classification, which can be built using several IoT and deep learning techniques [33]. Furthermore, self-monitoring systems can be seen as valuable components in the IoMT system such as weight and activity monitoring in diet, cardiovascular fitness, heartbeat, and nutrition planning programs [34,35,36].

Recently, the IoMT technologies have been evolving with the development of the artificial intelligence field, especially with the breakthrough of DL algorithms [18]. As a result, DL has enhanced both the specialist and the patient experience in the IoMT ecosystem providing accurate and fast diagnosis reports and helping the early prevention of disease spread. For instance, Rodrigues et al. [37] developed a vital healthcare system based on DL techniques, such as transfer learning, to classify skin lesions. Han et al. [38] investigated using DL techniques to process CT scan images and perform lung and stroke region segmentation. The authors established a communication channel with the patient relying on IoT technologies to provide diagnosis reports and consultations. Bianchetti et al. [39] proposed an automated ML system using tumour histotypes of dPET (dynamic positron emission tomography) data for adenocarcinoma lung cancer classification. Hossen et al. [40] developed a framework based on a federated learning approach and a convolution neural network (CNN) to classify human skin diseases and preserve data privacy.

Unlike fully automated DL systems, the IoMT still relies on the medical expert’s intervention to validate the results generated by a DL model or assess the accuracy of the DL model. However, DL models have shown a remarkable and accurate performance in many medical applications where they can help in decision-making and the early detection of infectious diseases from big data. Thus, developing a robust DL model to perform a specific task is vital to provide the patients with the best medicament and control the disease in its early stages [41].

### 2.2. Transfer Learning on Medical Images

In recent years, pretrained models for different applications have outperformed the regular learning process and training models from scratch. Thus, the performance on various applications has increased, and the learning time has been reduced [42]. The transfer learning process aims to transfer the knowledge learned while solving specific tasks to a new related task. For instance, Cheplygina et al. [43] addressed the use of transfer learning and different learning approaches in the medical field to perform medical image analysis. Transfer learning can be applied while fine-tuning all or specific layers to adapt the previously learned knowledge to the new related task. For instance, Ayan and Ünver [44] fine-tuned two pretrained models, including Xception and VGG16, trained on a large set of images from the ImageNet dataset. The fine-tuned models were used to detect pneumonia in chest X-ray images where the VGG16 exceeded the Xception model in terms of detection accuracy.

The ability to extract features from the VGG and ResNet models using bilinear classification techniques combined with SVM classifiers yielded the best results on several test sets [45]. A combination of data-driven approaches and InceptionV3 was used to train roughly 13W dermatology images, with findings on the testing set comparable to those of physicians [46]. Skin lesion segmentation was utilized to categorize melanoma in the ISBI-2016 skin lesion analysis towards cancer diagnosis [47]. As a result of this, the final classification had to be performed step by step. Multiple CNNs employing dynamic pattern training were used to simulate cancer intraclass conflict and associated noise interference in [48]. Kawahara et al. [49] decided to employ a pretrained CNN to identify skin images throughout their entire dataset rather than starting from scratch with randomly initialized parameters. After that pretraining, the CNN’s number of training rounds was considerably decreased, and the accuracy percentage for five classes was 84.8%. Lopez et al. [50] applied a deep learning method for early detection. It was developed using an adapted VGGNet design and a transfer learning technique. A sensitivity value of 78.56% was achieved using the ISIC archive dataset using the developed model. The performance of a CNN model for detecting lesions was tested using a dataset that was both extended and unaugmented in [51]. The researchers noted that deep learning approaches could be practical, and more data had to be collected. In addition, the network performed better on the additional dataset than other models. Yu et al. [47] implemented a very deep residual network-based multistage model for automatically detecting melanomas in dermoscopy images. They merged VGG and ResNet networks with the SVM classifier to improve the model’s detection performance. Zhang et al. [52] developed a deep synergic learning (SDL) model based on multiple deep CNNs in parallel with a sharing strategy for mutual learning. The authors validated the model’s performance on the ImageCLEF and ISIC datasets for medical image classification tasks.

Most of the well-known pretrained models in computer vision are based on convolution blocks, such as Inception, MobileNet, ResNet, DenseNet, and EfficientNet [53]. Furthermore, Transformer-based pretrained models were first established for language modelling and have been widely adopted for computer vision tasks. Transformer-based pretrained models benefit from the attention mechanism to learn contextual feature representation. For instance, ResViT [54] is a residual Vision-Transformer-based model for medical image tasks. ResViT synthesizes multimodal MRI and CT images in an adversarial learning process that relies on residual convolutional and transformer building blocks.

### 2.3. Medical Images Classification Using FS Optimizers

Currently, metaheuristic (MH) optimization techniques are applied to find solutions for different optimization problems. Those MH techniques provide a set of solutions rather than a single answer, supporting them in efficiently exploring the search space. Thus, they provide better results than traditional optimization approaches [55].

In the same context, Ravi K Samala et al. [56] presented an approach to the multilayered pathway used to predict breast cancer. They developed a two-stage approach consisting of transfer learning and determining features, respectively. To train pretrained CNNs, ROIs from large lesions were used. A random forest classification was developed based on the learned CNN. A genetic algorithm (GA) was used to select the relevant features. Silva et al. [20] optimized the hyperparameters of a CNN using PSO for the false-positive reduction in CT lung images.

Shankar K. et al. [57] developed the grey wolf optimization (GWO) technique for Alzheimer’s disease using brain imaging analysis. Then, a CNN was used to extract the features from the retrieved images. Goel et al. [58] developed an OptCoNet as an optimized CNN architecture for recognizing COVID-19 patients as having pneumonia or not. The GWO was used to determine the parameters of the convolution layer. To improve architectures for denoising images, Mohamed et al. [59] developed an enhanced version of firefly algorithms (FFA) to categorize the images as abnormal and normal. There was a significant enhancement in performance as a result of this adjustment. The diagnosis of melanoma was improved using the whale optimization algorithm (WOA) and levy flight [60]. These methods have some limitations, such as premature convergence, primarily when worked in a large search space [61]. These limitations have a negative impact on the prediction performance, especially in the IoMT environment. Therefore, the main objective of this paper was to determine the best solutions to improve the convergence rate by reducing the number of selected features.

As part of our developed study, to overcome these problems, transfer learning is integrated with metaheuristic optimization to build the IoMT framework. The qualities of this framework enable excellent performance and affordable computing expenses and address the financial concerns discussed earlier. Treating and detecting infections in or out of the clinic is essential. In order to use the IoMT system, all we need is an internet-connected device and a digital copy of the examination. The service’s quick reply allows for meaningful data throughout a session.

**Table 1:** The literature review on selected datasets.

DS	Model / Source	Methodology
ISIC-2016	CUMED [47]	Integrating a fully convolutional residual network (FCRN) and other very deep residual networks for classification.
	BL-CNN [45]	Combining two different types of deep CNN (DCNN) features as local and global features, using deep ResNet for the global features and a bilinear (BL) pooling technique to extract local features.
	DCNN-FV [62]	Integrating a ResNet method and a local descriptor encoding strategy. The local descriptors were based on a Fisher vector (FV) encoding to build a global image representation.
	MC-CNN [52]	Using multiple DCNNs simultaneously and enabling them to mutually learn from each other.
	MFA [63]	Cross-net-based combination of several fully convolutional were suggested. Used multiple CNNs for selecting semantic regions, local color and patterns in skin images. The FV was used to encode the selected features.
	FUSION [64]	MobileNet and DenseNet were coupled to boost feature selectivity, computation complexity, and parameter settings.
PH2	ANN [65]	A decision support system mad a doctor’s decision easier utilizing four distinct ML algorithms, where the artificial neural network (ANN) achieved the best performance.
	DenseNet201-SVM [66]	U-Net was used with spatial dropout to solve the problem of overfitting, and different augmentation effects were applied on the training images to increase the data samples.
	DenseNet201-KNN [37]	Combined twelve CNN models as resource extractors with seven different classifier configurations, which the greatest results obtained using the DenseNet201 model with a KNN classifier.
	ResNet50-NB [67]	A ResNet model was applied to map images and learn features through TL. The extracted features were optimized using a grasshopper optimization algorithm with a naïve Bayes classification.
Blood-Cell	CNN-SVM [68]	A CNN with SVM-based classifiers with features derived by a kernel principal component analysis of the intensity and histogram data was able to classify images.
	CNN [69]	An SVM and a granularity feature were used to detect and classify blood cells independently. CNNs were utilized to automatically extract high-level features from blood cells, and these features were then used to identify the other 3 types of blood cells using a random forest.
	CNN-Augmentation [70]	The extraction and selection of features, as well as the classification of white blood cells, were all automated. A DL approach was used to automate the entire procedure with CNNs for binary and multiclass classification.

## 3. Background

Improved deep learning for extracting features and two feature selection algorithms, the arithmetic optimization algorithm and the hunger games search are all presented in the following.

### 3.1. Enhanced Deep Learning

It has been shown that DL methods are effective in various tasks, such as the categorization [71] and segmentation of images and object identification [72]. There is still much to know about the difficulties of these activities, particularly regarding the quality and effect of the acquired representations. Many DL architectures and learning methods have been developed during the last decade. With its many topologies, layouts, settings, and training procedures, the CNN is among the most studied DL models. Instead of conventional convolution operation, depthwise separable convolutions may be used on embedded devices or edge apps since they replace the existing convolutions. In order to overcome the drawbacks of conventional convolution operation, numerous DL models have adopted the idea of depthwise separable convolutions, such as EfficientNet [73]. The depthwise separable convolutions differ from conventional convolution operations in that they are applied individually to each input port. As a result, the models are operationally affordable and can be learned using lower parameters and little training time. MobileNetV3 [74] is available in two structures based on the model size: MobileNetV3-large and MobileNetV3-small. Compared to MobileNetV2, the MobileNetV3 structure is intended to reduce delay and improve accuracy. MobileNetV3-large, for example, increased accuracy by 3.2% over MobileNetV2 while decreasing latency by 20%. The NetAdapt method was used to find the best network topology and kernel dimensions for the convolution layer on MobileNetV3. The MobileNetV3 structure comprises the following fundamental components: a depth-separable convolution operation with a specific convolution kernel, a batch normalization, and an activation function. Next, the depthwise-separable, fully connected layer’s mutual information calculations and the retrieval of hidden units use 1 × 1 convolutions. Third, a global average pooling makes feature maps more manageable regarding their spatial dimension. Furthermore, by using an inverted residual block [75], we may avoid the bottlenecks caused by the residual skipped-connection method. These blocks make up the inverted residual one: (a) using the 1×1 extension and convolutions, as well as the depthwise convolution kernels of size 1×1, for more complicated representations and to reduce model computations; (b) a convolutional layer with depth separation; (c) a method for retaining a skip connection. In addition, the squeeze-and-excite (SE) block [74] can be used to choose the appropriate features channel by channel. Finally, a rectified linear unit (ReLU) and the h-swish activation function are interchangeable terms for the same thing, the activation function.

### 3.2. Arithmetic Optimization Algorithm

The arithmetic optimization algorithm (AOA) [76] is an MH technique that depends on essential functions to find the optimal solution. Like other MH techniques, it begins with a randomized number of candidate alternatives (*X*) and the best-obtained or nearly optimal solution. For the AOA to begin functioning, the search stage should be selected first (i.e., exploration or exploitation). In the following search stages, the maths optimizer accelerated (MOA) is used and defined as in Equation (1).

(1) $$MOA\left(t\right)=Min+t\times \left(\frac{Max-Min}{T}\right)$$

The variable *t* denotes the current repetition and ranges from one to the maximum allowable number of epochs (*T*). The terms indicate the accelerating function’s lowest and greatest values, Min and Max.

To discover an ideal option, AOA’s exploration agents examine the research scope at random locations across multiple areas, using two primary search methods (the divide technique and the multiply technique described in Equation (2).

(2) $$x_{i,j}\left(t+1\right)=\left\{\begin{array}{c} Xb_{j}÷\left(M_{OP}\right)\times \left(UL_{j}\times \mu +LB_{j}\right),r2>0.5\\ Xb_{j}\times M_{OP}\times \left(UL_{j}\times \mu +LB_{j}\right),Otherwise \end{array}\right.$$

where $UL_{j}=UB_{j}-LB_{j}$. In this scenario, $x_{i}\left(t+1\right)$ represents the *i*th solution during the next repetition, $x_{i,j}\left(t\right)$ represents the *j*th location of the *i*th solution in the latest iteration, and $Xb_{j}$ represents the *j*th place in the optimal method thus far. ϵ is a tiny integer number. The *j*th location’s minimum and maximum bounds are denoted by $UB_{j}$ and $LB_{j}$, respectively. The μ=0.5 process parameters regulate the search behaviour.

(3) $$M_{OP}\left(t\right)=1-+\frac{t^{1/\alpha }}{T^{1/\alpha }}$$

where $M_{OP}\left(t\right)$ in Equation (3) represents the probability of the maths optimizer ($M_{OP}$). The current iteration is represented by *t*, while the total number of iterations is represented by (*T*). The exploitation accuracy across iterations is defined by the sensitivity parameter α=5.

It is necessary to do this stage of exploitation by only researching if r1 is less than the existing MOA(t) quantity (see Equation (1)). In AOA, the exploitation operators (subtraction and addition) discover the research scope intensely across several populated areas and methods to produce a solution based on two primary search techniques (i.e., subtraction and addition) that are modelled in Equation (4).

(4) $$x_{i,j}\left(t+1\right)=\left\{\begin{array}{c} Xb_{j}-M_{OP}\times \left(UL_{j}\times \mu +LB_{j}\right),r3>0.5\\ Xb_{j}+M_{OP}\times \left(UL_{j}\times \mu +LB_{j}\right),Otherwise \end{array}\right.\;$$

### 3.3. Hunger Games Search

The hunger games search (HGS) algorithm was developed by [77] as an optimization technique that resembles organismal biology. As a result of the HGS, a creature’s capacity to use hunger as a physiological incentive for all of these things is one of its most distinguishing features. HGS mathematical modelling begins with a population of *N* alternatives *X* before obtaining $Fit_{i}$ estimates for each alternative’s fitness function. The modernization step is instead carried out using the given formula in Equation (5).

(5) $$X=\left\{\begin{array}{c} X\left(t\right)\times \left(1+rand\right),\;r_{1}<l\\ W_{1}\times Xb+R\times W_{2}\times X_{bi},\;r_{1}>l,\;r_{2}>E\\ W_{1}\times Xb-R\times W_{2}\times X_{bi},\;r_{1}>l,\;r_{2}<E \end{array}\right.$$

where $X_{bi}=\left|Xb-X\left(t\right)\right|$. The two variables $r_{1}$ and $r_{2}$ represent random numbers, and the parameter rand produces random numbers from a normally distributed set. The parameter *R* determines the search area and may be dependent on the number of rounds as defined in Equation (6).

(6) $$R=2\times s\times rand-s,\;\;\;s=2\times \left(1-\frac{t}{T}\right)$$

where *E* indicates the parameter, which is specified as in Equation (7).

(7) $$E=\mathrm{sech}\left(\left|Fit_{i}-Fit_{b}\right|\right)$$

$Fit_{b}$ indicates the fitness function’s highest value, whereas Sech denotes the hyperbolic value, defined as in Equation (8).

(8) $$\mathrm{sech}\left(x\right)\;=\frac{2}{e^{x}-e^{-x}}.$$

Additionally, $W_{1}$ and $W_{2}$ are the hunger weights from Equations (9) and (10).

(9) $$W_{1}=\left\{\begin{array}{c} H_{i}\times \frac{N}{SH}\times r_{4},\;r_{3}<l\;\\ 1,\;\;\;\;\;r_{3}>l \end{array}\right.$$

(10) $$W_{2}=2\left(1-e^{\left(-|H_{i}-SH|\right)}\right)\times r_{5}$$

SH represents the solution’s hunger-experiencing accumulation, and the parameters SH correlate to $r_{3},\;r_{4}$ and $r_{5}$ being random integers with ranges in the interval [0, 1], as follows:

(11) $$SH=\sum_{i}H_{i}$$

(12) $$H_{i}=\left\{\begin{array}{c} 0,\;\;\;\;\;\;\;Fit_{i}=Fit_{b}\\ H_{i}+H_{n},\;\;\;otherwise \end{array}\right.$$

where $H_{n}$ represents the new hunger, and it is formulated as:

(13) $$H_{n}=\left\{\begin{array}{c} LH\times \left(1+r\right),\;\;\;\;\;\;TH<LH\\ TH,\;\;\;otherwise \end{array}\right.$$

(14) $$TH=2\frac{Fit_{i}-Fit_{b}}{Fit_{w}-Fit_{b}}\times r_{6}\times \left(UB-LB\right)$$

Moreover, there is a lower value provided by $Fit_{w}$ for the fitness function; in addition, $r_{6}\in \left[0,1\right]$ is a randomised number that indicates if hunger has positive or negative effects based on various variables.

## 4. Developed Approach

To accomplish our approach, we created a 6G-enabled IoMT framework. It is capable of transmitting data quicker than a 5G-enabled system. In bandwidth, 6G may reach microseconds, significantly improving its speed over 5G’s milliseconds [78]. Furthermore, 6G enables real-time broadcast and processing better quality images and assists artificial intelligence in achieving real-time broadcast and execution. Nevertheless, only low-latency and high-bandwidth wireless communication technologies can satisfy the developing requirements of DL and IoMT. Therefore, based on the 6G network and DL model concepts, we suggest incorporating the combined DL and FS optimizer algorithms presented in the following subsections into our 6G-enabled IoMT framework.

### 4.1. Feature-Extraction-Based Deep Learning

To identify and extract feature information, we utilized a transfer learning approach. Pretrained models for image recognition tasks are helpful because they speed up training and implication. Instead of building models from scratch, it is possible to fine-tune a few layers while the model’s weights are fine-tuned. We replaced the model’s top part with new layers for classification and feature extraction. MobileNetV3 was used as a core block for extracting features after fine-tuning its weights on different task-specific datasets.

MobileNetV3 was adjusted and trained to retrieve feature representations from input with a size equal to 224×224. The ImageNet data [75] were used to train the MobileNetV3 model and produce pretrained versions based on the model size (large or small). We used the dataset representing images of skin cancer, blood cells, and optical tomography to fine-tune the MobileNetV3-Large pretrained model. In our experiments, we replaced the MobileNetV3 model’s classification layer with two layers represented as 1×1 pointwise convolutions to extract the image representations and fine-tune the model for the classification task.

The 1×1 pointwise convolution is often used to categorize and extract features that have similar applications to those of multilayer perceptrons (MLPs). After fine-tuning the MobileNetV3 layers, we fed the extracted features to a 1×1 pointwise convolution which learned task-specific features. The MobileNet3 core layers are a combination of inverted residual blocks stacked sequentially. Each inverted residual block consists of several components derived from the MobileNetV2 structure, including a 1×1 expansion convolution, a depthwise-separable convolution, a squeeze-and-excite block, a 1×1 projection convolution, and a skip-connection mechanism. Furthermore, a kernel of size 3×3 is used in the depthwise-separable convolution with an activation function which can be placed in the following order (3×3Conv)→(BN)→(ReLU/h−swish)→(1×1Conv)→(BN)→(ReLU/h−swish). A depth-separable fully connected layer with various nonlinearity variables, including hard swish (h-swish) or ReLU, may be included in each construction block. These functions are described in Equations (15) and (16).

(15) ReLU(x)=max(0,x)

(16) $$\begin{array}{c} h-swish(x)=x\times \sigma (x) \end{array}$$

where σ(x) specifies the piecewise linear difficult analogue functional, where $\sigma \left(x\right)=\frac{ReLU6(x+3)}{6}$. The output of the 1×1 pointwise convolution placed before the classification layer (1×1 pointwise convolution) is the feature extraction block that generates the learned image embeddings during the network training and fine-tuning. Each extracted image embedding is represented with a 128-feature vector. The developed model was trained on each dataset for 100 epochs with a batch size of 32 with an early stopping strategy (20 epochs). The RMSprop algorithm, with the learning rate of $1\times 10^{-4}$ was applied to modify the model’s weight and bias values. For this reason, we employed a dropout layer and data augmentation with randomized horizontal flips, randomized crops, colour jitters, and periodic vertical flips to counteract the model’s overfitting problem. The Pytorch framework was used to implement the model, and the training was conducted on an Nvidia RTX1080 GPU.

### 4.2. The Developed FS Algorithm

This article aims to provide a novel technique for enhancing the efficiency of the arithmetic optimization algorithm (AOA). This was achieved by using the operators of the hunger games search (HGS) algorithm. Whenever the AOA could not discover the optimal solution within a specified iteration, a much more effective searching focused on the HGS was applied to enhance the exploration ability. The HGS enhanced the capacity to do global and regional searches concurrently.

**Figure 1:**
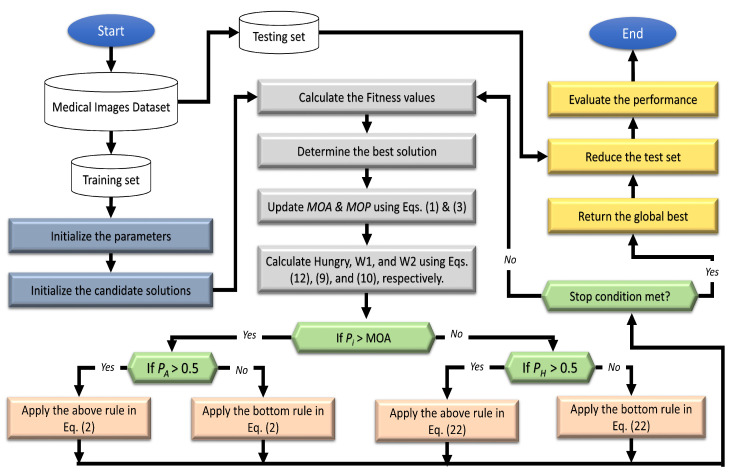
Flowchart showing the developed FS algorithm.

The basic steps of the FS technique, called AOAHG, are shown in Figure 1. The initial stage in the developed AOAHG was to create the set of *N* agents *X*, reflecting the FS challenge solutions. This procedure was obtained by using the following formula:

(17) $$X_{i}=rand*\left(U-L\right)+L,\;i=1,2,\ldots ,N,\;j=1,2,\ldots ,Dim$$

The term Dim denotes the number of features. As a result, the available dimensionality was restricted to values between *U* and *L*. We used the following equation to obtain the binary form of each $X_{i}$:

(18) $$BX_{ij}=\left\{\begin{array}{cc} 1 & if\;X_{ij}>0.5\\ 0 & otherwise \end{array}\right.$$

As a further step, we calculated the fitness value for $X_{i}$ as in Equation (19), based on its binary form $BX_{i}$.

(19) $$Fit_{i}=\lambda \times \gamma_{i}+\left(1-\lambda \right)\times \left(\frac{|BX_{i}|}{Dim}\right),$$

In this case, the proportion of features associated is denoted as $\left(\frac{|BX_{i}|}{Dim}\right)$. $\gamma_{i}$ is the validation loss of the SVM. In general, the SVM is often applied because it is more reliable and has fewer parameters than other classifiers. The value of the parameter λ balances the ratio between the accuracy of a classifier’s predictions and the selection of features.

The following procedure was used to adjust a solution $X_{i}$ by using either the HGS or AOA operators. This was accomplished through the use of the probability $P_{i}$ associated with each $X_{i}$. While the HGS may take longer, it was used if the probability of $P_{i}$ was less than the MOA, as defined by the given equations:

(20) $$X_{ij}=\left\{\begin{array}{cc} X_{ij}^{A} & if\;P_{i}>MOA\\ X_{ij}^{HG} & otherwise \end{array}\right.$$

where MOA is specified in Equation (1). The value of $X_{ij}^{A}$ is updated using the operators of the AOA as described in Equation (2).

(21) $$X_{ij}^{A}=\left\{\begin{array}{cc} The\;first\;rule\;of\;Equation(2) & if\;P_{A}>0.5\\ The\;second\;rule\;of\;Equation(2) & otherwise \end{array}\right.$$

where $P_{A}\in \left[0,1\right]$ is a random sample variable that is utilised to keep the AOA operators comparable throughout solution updates.

The HGS operators were then applied on the upgraded population of *X*. The following formula yielded $X_{ij}^{HG}$:

(22) $$X_{ij}^{HG}=\left\{\begin{array}{cc} W_{1}\times X_{ij}-R\times W_{2}\left|X_{ij}-X\left(t\right)\right| & ifP_{H}>0.5\\ W_{1}\times X_{ij}+R\times W_{2}\left|X_{ij}-X\left(t\right)\right| & otherwise \end{array}\right.$$

where $W_{1}$ and $W_{2}$ are defined in Equations (9) and (10), respectively. If $P_{H}$ was greater than 0.5, the first HGS rule was applied; otherwise, the second HGS rule was applied.

Additionally, the search space [U,L] was dynamically changed throughout the finding process as follows:

(23) $$L_{j}=min\left(X_{ij}\right)$$

(24) $$U_{j}=max\left(X_{ij}\right)$$

The next stage was to determine if the closure conditions were met, and if so, the optimum solution was given. If this happened, the upgrade procedure was repeated from the beginning. The suggested AOAHG’s pseudocode is given in Algorithm 1.
**Algorithm 1** Pseudocode of the developed AOAHG algorithm1:Initialize the parameters.2:Split the dataset into training and testing sets after extracting the features.3:Initialize the number of solutions (*N*).4:**repeat**5:      Determine the value of the fitness function.6:      Find the best solution.7:      Update the MOA value using Equation (1).8:      Update the $M_{OP}$ value using Equation (3).9:      Calculate the hunger weight of each position using Equations (9) and (10).10:    Enhance Hi using Equation (12).11:    **for** i=1 to *N* **do**12:        **for** j=1 to Positions **do**13:              Generate a random values in [0, 1] ($P_{i}$, $P_{A}$, and $P_{H}$).14:              **if** $P_{i}$ > MOA **then**15:                     Position limitations can be adjusted for new seeds.16:                     **if** $P_{A}$ > 0.5 **then**17:                            Update *i*th solutions’ positions by the first rule in Equation (2).18:                     **else**19:                             Update *i*th solutions’ positions by the second rule in Equation (2).20:              **else**21:                     **if** $P_{H}$ > 0.5 **then**22:                             Update *i*th solutions’ positions by the first rule in Equation (22).23:                     **else**24:                             Update *i*th solutions’ positions by the second rule in Equation (22).25:**until** The iteration (*t*) criterion has been met.26:Return the best solution.

### 4.3. Sixth-Generation-Enabled IoMT Framework

**Figure 2:**
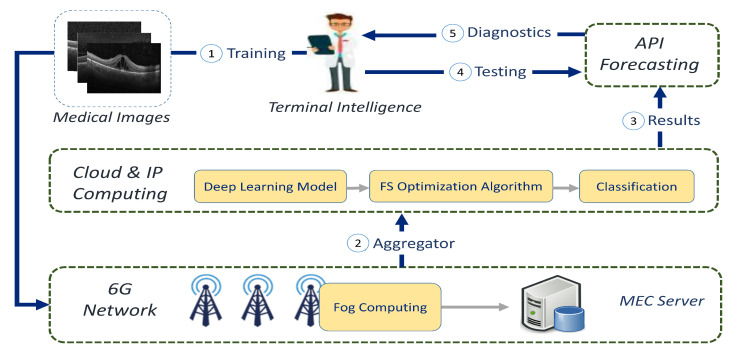
The suggested 6G-enabled IoMT framework diagram.

The suggested 6G-enabled IoMT architecture is shown in Figure 2. The terminal intelligence of the IoT first collected diagnostic images, and if the expert’s aim was to learn the framework, the input images could be transmitted through a 6G network. Then, the data collected from the multiaccess edge-computing servers could be uploaded to a cloud computing service.

The three primary processes were still in place in cloud computing. In the first stage, the DL design’s features were retrieved, as discussed in Section 4.1. As a second stage, we used the modified AOA depending on an HGS (AOAHG) to select the significant features, as illustrated in Section 4.2. Finally, once the classifier had been learned, it could be distributed across several API forecasting/prediction nodes, saving on transmission fees.

On the other hand, if the user’s goal was to test the case/disease of the collected image, the test pattern in the API prediction/forecasting tools was employed. API forecasting enabled the system’s authorized training product to forecast anything without retraining, saving time and reducing internet traffic. Finally, the sender/specialist was given the last diagnostic and several evaluation metrics such as accuracy, F1-score, and others to back up the system’s forecasts.

The time complexity of the developed method depended on the AOAHG and MobileNetV3. The complexity of the developed AOAHG method was represented as O(N×(T×D+N+1) where N, T, and *D* are the number of solutions, iterations, and dimensions, respectively. In addition, MobileNetV3 had around 3 million trainable parameters.

## 5. Experimental Studies and Results

### 5.1. Dataset

**Figure 3:**
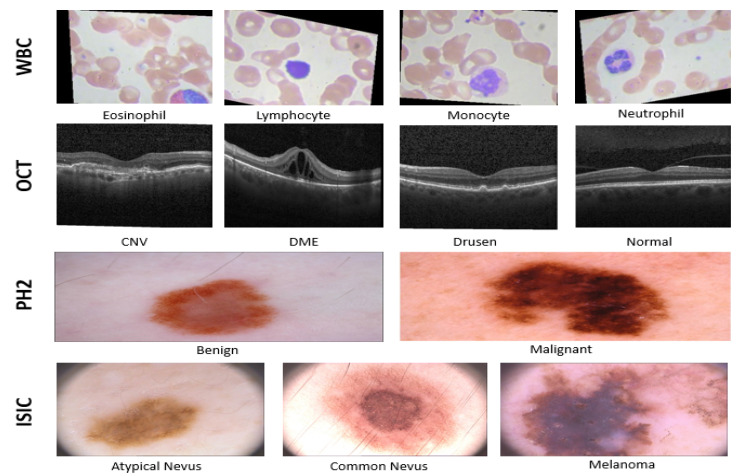
Sample of images from: ISIC, PH2, WBC, and OCT datasets.

Four medical datasets were employed for our experimental assessment, including a white blood cells (WBC) dataset, retinal optical coherence tomography (OCT) images, and skin images to identify malignant ones. To perform skin cancer classification, two datasets of dermatoscopic images were used: PH2 [79] and ISIC-2016 [80]. Figure 3 depicts a sample of images from the tested datasets. 

#### 5.1.1. WBC Dataset

The accessible data utilized in this research were classified into four types, as described in [81], and included the following: eosinophil, lymphocyte, monocyte, and neutrophil. The WBC dataset contains microscopic images of 3120 eosinophils, 3103 lymphocytes, 3098 monocytes, and 3123 neutrophils. Each picture has a resolution of 320 × 240 pixels and a depth of 24 bits. Furthermore, the dataset was divided into two parts: 80% for training and 20% for testing. To be more specific, the training set had 2496 eosinophils, 2484 lymphocytes, 2477 monocytes, and 2498 neutrophils, while the testing set contained 620 from monocytes, 624 from neutrophils, also, 623 from eosinophils and lymphocytes.

#### 5.1.2. OCT Dataset

In this section, we introduce the description of the OCT dataset, which consists of 84,484 OCT B-scans obtained using 4686 patients (collected at the Shiley Eye Institute of the University of California, San Diego (UCSD)). These images are categorized into four classes, DME, CNV, drusen, and normal, which contain 8866, 37,455, 11,598, and 26,565 images, respectively. Additionally, this dataset includes 968 test images and 83,516 training images. For the training, 37,213, 11,356, 8624, and 26,323 images were used from each class, respectively, and for the testing set, we used all images from each class.

#### 5.1.3. PH2 Dataset

A sample size of 200 dermoscopy images was included in the PH2 dataset, comprising 80 common nevus, 80 atypical nevus, and 40 melanoma. The data were split into 85% training and 15% testing sets.

#### 5.1.4. ISIC Dataset

In all, 1179 samples were included from the ISIC-2016 dataset, which was divided into two categories including benign and cancerous. The ISIC-2016 dataset contains 248 images of malignant tumours and 1031 images of benign tumours. Furthermore, the data were divided into 70% and 30% training and testing sets, respectively. For the training, we used 173 malignant and 727 benign images, whereas for the testing, we used 75 malignant and 304 benign images.

To assess the efficiency of the developed method for classifying medical images, the recall, precision, balanced accuracy, accuracy, and F1-score were used.

### 5.2. Experimental Results and Discussion

This section summarises the results of the experiments conducted to evaluate the efficiency of the developed 6G-IoMT approach. We assessed our developed FS method against other FS based on MH approaches, including the Aquila optimizer (AO) [82], PSO, GWO, moth-flame optimization (MFO) [83], bat algorithm (BAT) [84], Archimedes optimization algorithm (ArchOA) [85], chaos game optimization (CGO) [86], hunger games search (HGS) [77], and arithmetic optimization algorithm (AOA) [76]. After that, extreme gradient boosting (XGB), the K-nearest neighbours (KNN), random forest (RF), and support vector machine (SVM) classifiers were assessed against each other. All tests used a population size of 50 and 20 iterations. The other parameters were set according to the original implementation.

#### 5.2.1. Results of FS Methods

Results from the ISIC-2016 dataset and PH2 dataset can be found in Table 2. Table 3 contains the findings from the WBC dataset and OCT dataset.

From Table 2, the SVM-based AOAHG provided better results than other approaches on the ISIC-2016 dataset. The accuracy of the AOAHG algorithm using the SVM was 87.34%, representing the best efficiency, followed by MFO, which achieved the second rank with 86.54%. ArchOA, AOA, and HGS followed the previous two algorithms (AOAHG and MFO). The BAT and CGO algorithms, with 86.02%, followed the preceding methods. The algorithms that followed were the PSO (85.75%), AO (85.49%), HGS (84.96%), and GWO (84.43%) algorithms. For the value of precision, the developed AOAHG method achieved 86.53%, followed by the MFO with an 85.60% accuracy.

The results of the recall of the AOAHG method were better than others. The developed algorithm was followed by MFO, which had a success rate of 85.54%. The ArchOA and AOA all had the same value of recall, that is, 86.28%. For the vote, the PSO obtained 86.02%, and both CGO and BAT achieved 85.75%. Finally, the GWO algorithm had the worse outcome at 84.43%. The presented AOAHG method outscored the other methods with an F1-score of 86.47%. The AOA and ArchOA obtained the exact value of 85.73%. Next, MFO, CGO, BAT, and AO had an F1-score of 85.57%, 85.50%, 85.00%, and 84.86%, respectively.

For the PH2 dataset, Table 2 illustrates that the AOAHG method significantly improved the determination of features when using an SVM classification method; this was evident across all metrics. According to the accuracy measure, AOAHG correctly classified 96.43% of the testing samples when an SVM was used. In addition, these results were significantly different from the accuracy of other FS approaches. Moreover, the AOAHG had the better precision value of any SVM methods at 96.44%, the highest of any other optimizer algorithm. AOA, CGO, BAT, ArchOA, MFO, GWO, and PSO placed second. Following these optimizers were the AO and the HGS methods, which achieved an 96.70% accuracy. As a further analysis, the recall metric for the SVM classification model was 96.43% for AOAHG, which indicated that the developed method had the maximum effectiveness. The developed AOAHG method was the best optimizer based on the F1-score with 96.43%. The PSO, GWO, MFO, ArchOA, AO, BAT, HGS, CGO, and AOA approaches had an F1-score of nearly 96.07%. Furthermore, the presented AOAHG method had the highest balanced accuracy value, nearly 97.02%. These algorithms came in second place for all the other optimization algorithms with 96.73%. Nevertheless, when these ten optimizers were combined with the KNN, XGB, and RF classifiers, the outcomes had the poorest overall performance measures compared to those of the SVM classification algorithm.

Table 3 shows the comparison between the AOAHG approach and other optimizers using the white blood cell dataset. Based on the results, the AOAHG algorithm based on an SVM provided a better accuracy (nearly 88.62%) than the other algorithms. The AOA was second with an accuracy of 88.58%. Then, the MFO and HGS techniques had the same outcome (i.e., 88.54%), and both the CGO and GWO approaches obtained the same accuracy value, 88.50%. In addition, from those results, it can be noticed that the ArchOA and AOA had the worst score at 88.26%.

Moreover, the recall values of AOAHG were better than those of the other methods. The AOA, HGS, and MFO methods all had similar recall values (i.e., around 88.54%). Finally, the AO and ArchOA had a worse outcome of 88.26%. The developed AOAHG technique outperformed other methods according to the F1-score, which was 88.80%. The AOA was second, with 88.76%, and the ArchOA obtained the worst F1-score value of 88.44%. Based on the results of the balanced accuracy, the AOAHG algorithm provided better results with an accuracy of 88.62%. Similarly, according to the balanced accuracy, the AOA was ranked second (88.58%).

Table 3 shows the results of the algorithms applied on the OCT dataset. From those results, it can be noticed that the AOAHG method was better than the other optimization methods. The best performance based on the accuracy measure was the AOAHG approach using the SVM with a 99.69% accuracy. At the same time, the CGO and HGS methods were ranked second with an accuracy 99.59%. Based on the precision value, the developed AOAHG approach achieved a score of 99.69%. The CGO and HGS algorithms followed with a precision of 99.59%. The precision of the AOA, BAT, ArchOA, MFO, and GWO algorithms was 99.40%. On the other side, the AOAHG approach had the highest recall measure performance of any SVM classifiers at 99.69%. Coming in second were HGS and CGO, which both had a recall of 99.59%. Five optimizers had a common recall value of almost 99.38%: GWO, MFO, ArchOA, BAT, and AOA. On the other hand, PSO and AO performed the worst at 99.28%. Our new algorithm (i.e., AOAHG) outperformed earlier ones with 99.69% on the F1-score metric. Following that, CGO and HGS both obtained 99.59% for the F1-score. Nevertheless, that was not the end of it. With a result of 99.28%, AO and PSO came in dead last in the competition. The AOAHG algorithm had the most excellent actual quality, with a balanced accuracy of 99.69%. HGS and CGO obtained 99.59% and came in second. Following that, AOA, BAT, ArchOA, MFO, and GWO had a balanced accuracy of 99.38%. However, PSO and AO had the worst results, with a balanced accuracy of just 99.28%.

**Figure 4:**
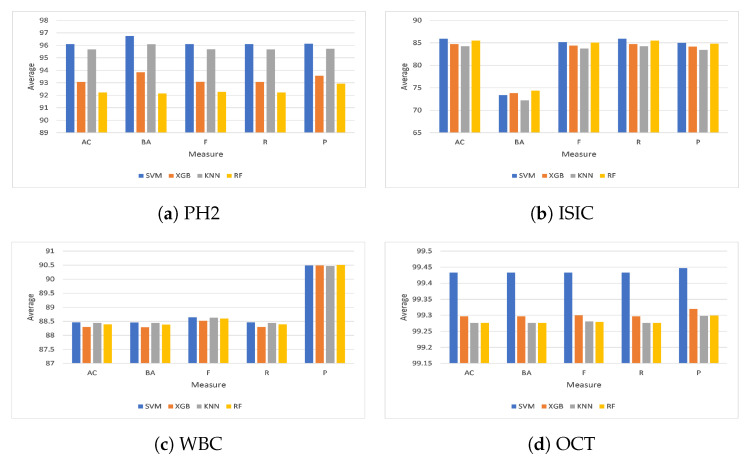
Average results from the four classifiers on the selected datasets.

From a different viewpoint, as shown in Figure 4, the average outcomes of the ten feature selection optimizers investigated on the four classifiers (i.e., SVM, KNN, RF, and XGB) on the four chosen datasets, PH2, ISIC, WBC, and OCT, are given in Figure 4. From Figure 4a it can be noticed that the overall average accuracy on the PH2 dataset was nearly 96.11% and 95.68% for the SVM and KNN, respectively. In addition, the overall balanced accuracy of the SVM classifier was the best (96.76%). It was followed by the KNN (96.10%), the XGB (93.84%), and the RF (92.14%) classifiers. Moreover, the best F1-score from the ten optimization techniques was obtained by the SVM at about 96.11%; the KNN was second with 95.69%. Furthermore, the XGB outperformed the RF algorithm, with a success rate of 93.08% for XGB and 92.27% for RF, whereas the SVM was better than the other classifiers based on the recall value. Furthermore, the SVM achieved 96.11%, while the KNN classifier achieved 95.68%. Finally, the XGB and RF algorithms obtained 93.07% and 92.22%, respectively. In terms of precision measure, the SVM classification algorithm delivered superior results compared to the KNN, XGB, and RF classifiers, with 95.73%, 93.56%, and 92.93%, respectively.

As shown in Figure 4b, the average accuracy of the ten optimization techniques on the ISIC dataset using the SVM was 85.91%; the RF algorithm took second place with 85.49%. Furthermore, the XGB achieved 84.75%, outperforming the KNN, which achieved 84.28%. Moreover, the RF was better than the other classifiers in terms of balanced accuracy. To be more specific, the RF achieved 74.38%, while the XGB achieved 73.82%. Finally, the SVM and KNN algorithms obtained 73.39% and 72.22%, respectively. Regarding the F1-score measure, the SVM classification algorithm delivered superior results compared to those of the RF, XGB, and KNN classifiers, with 85.04%, 84.39%, and 83.74%, respectively. Additionally, the overall average recall was approximately 85.91% for the SVM classifier, whereas the RF classifier came in second with 85.49%. XGB obtained a higher percentage (with 84.75%) compared to the KNN classification algorithm. In addition, the SVM classifier precision was the highest at 85.01%. It was followed by the RF (84.08%), the XGB (84.18%), and the KNN (83.46%).

As shown in Figure 4c, the SVM classifier achieved the highest accuracy on the WBC dataset with 88.46% followed by the KNN (88.44%), RF (88.39%), and XGB (88.30%) classifiers, respectively. Meanwhile, the BA metric of all optimization approaches outperformed the SVM classifier by 88.46% and the KNN classifier by 88.44%, respectively. The RF classifier surpassed the XGB classifier, with an accuracy equal to 88.39% for RF and 88.29% for XGB. The SVM classifier scored an average F1-score equal to 88.65%, whereas the KNN scored 88.63%. The RF obtained a higher F1-score (with 88.60%) than the XGB classification algorithm. The SVM classification algorithm delivered better results than the KNN, RF, and XGB classifiers, with a recall equal to 88.44%, 88.39%, and 88.30%, respectively. Additionally, the RF was better than the other classifiers based on the precision score. For instance, the RF achieved 90.51%, while the KNN and SVM achieved 90.49%. Finally, the XGB algorithm obtained 90.47%.

As shown in Figure 4d, the SVM classification algorithm delivered a superior average accuracy score on the OCT dataset compared to XGB, KNN, and RF classifiers, with 99.30%, 99.28%, and 99.28%, respectively. The average balanced accuracy was 99.43% for the SVM classifier, whereas the XGB classifier came in second with 99.30%. KNN obtained a lower average balanced accuracy (with 99.28%) than the RF classification algorithm. From a different perspective, the overall F1-score of the SVM classifier was the best (99.43%). It was preceded by the XGB (99.30%), the KNN (99.28%), and the RF (99.28%) classifiers. Meanwhile, the SVM scored a better recall than the other classifiers, equal to 99.43%, while the recall for XGB was 99.30%. The KNN and RF algorithms obtained 99.28%. Moreover, the SVM classifier achieved an average precision equal to 99.45%, followed by the XGB classifier with 99.32%. In addition, the KNN and RF classifiers achieved the same precision at 99.30%.

**Figure 5:**
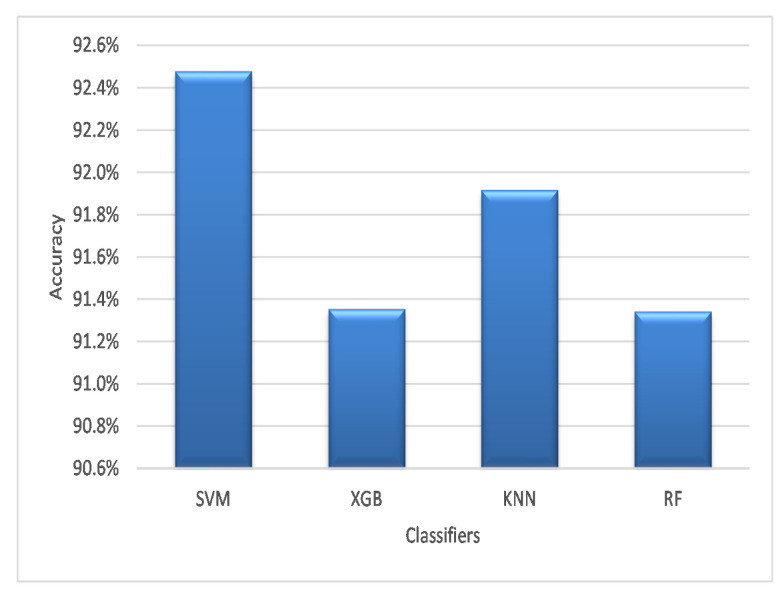
Average accuracy on the four classifiers.

Figure 5 presents the average accuracy of the experimented classifiers on the four datasets using different optimization strategies. As shown in Figure 5, the SVM showed a significantly better performance compared to the other classifiers in terms of accuracy. To be more precise, the SVM had an accuracy of 92.48%, while the KNN method had an accuracy of 91.92%. Finally, the XGB and RF algorithms achieved an accuracy of 91.35% and 91.34%, respectively.

**Figure 6:**
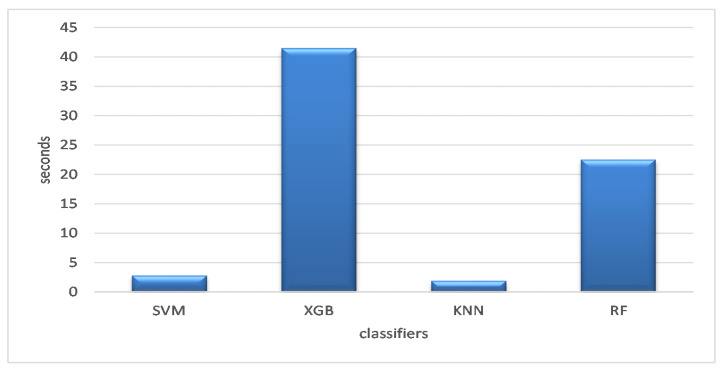
Average execution time of the classifiers across the datasets.

The whole operation took less time to finish than it did for a consumer, as shown in Figure 6, which displays the average execution time for the optimizers on the selected datasets. According to the findings, the KNN classification model was the fastest (i.e., less time), followed by the SVM classification algorithm, which required 2.7829 s to finish. In all, 22.5073 s were needed to complete the RF classification. The XGB required the longest duration at 41.4983 s.

**Figure 7:**
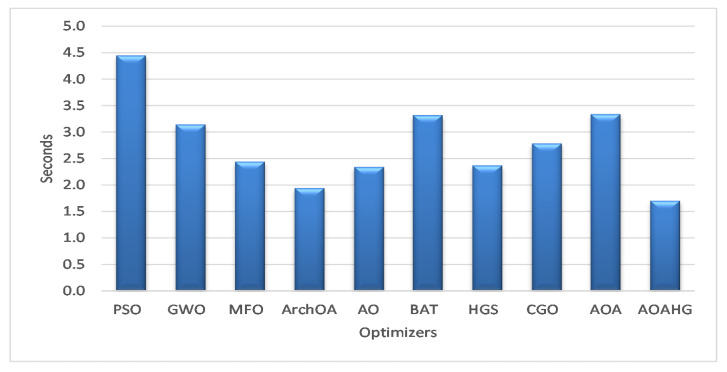
Average execution times of the SVM across the datasets.

For the four datasets, using the SVM classifier, the developed AOAHG and ArchOA took an average of 1.7031 and 1.9405 s to execute, as shown in Figure 7. Overall, these times were faster than those of other similar methods. The AO optimizer completed in 2.3397 s, while the HGS, MFO, CGO, GWO, BAT, and AOA optimizers completed in 2.3704 s, 2.4415 s, 2.7837 s, 3.1439 s, 3.3205 s, and 3.3388 s, respectively. For instance, the PSO algorithm achieved the longest execution time (4.4469 s).

**Figure 8:**
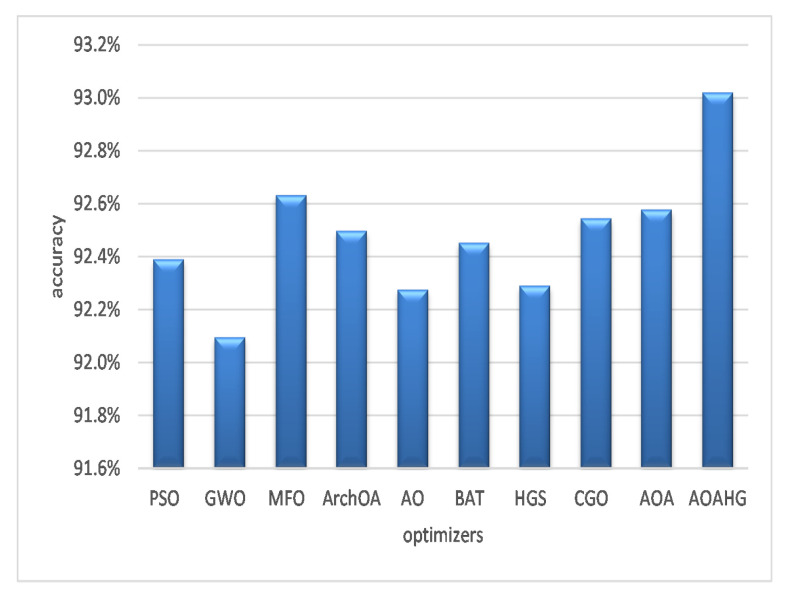
Average accuracy of the SVM across the datasets.

From a different viewpoint, Figure 8 illustrates each feature selection strategy on four datasets and the corresponding average accuracy. Using various optimizers, the SVM classifier outperformed the AOAHG technique on average with a 93.02% accuracy. The MFO method ranked second with an accuracy of 92.63%. The AOA outperformed the CGO, ArchOA, and BAT algorithm, averaging accuracies of 92.55%, 92.50%, and 92.45%, respectively. The PSO obtained an accuracy of 92.39%. These three optimizers produced the worst results, with an average balanced accuracy of 92.29% (HGS), 92.28% (AO), and 92.1% (GWO).

To summarise, for the ISIC-2016, PH2, WBC, and OCT datasets, the AOAHG algorithm alongside the SVM classifier obtained the best accuracy. In addition, our suggested method also produced the quickest results (i.e., the least execution time).

#### 5.2.2. Compared Methods

Other medical image categorization approaches are examined in this section to compare our developed method. Table 4 summarises the findings of many critical methods. Medical image categorization requires the development of highly accurate technologies. Comparing our approach to other models evaluated on the same datasets is critical. Table 4 compares the accuracy of various illness detection approaches using the ISIC, PH2, WBC, and OCT datasets.

The ISIC dataset was used to evaluate various skin cancer detection techniques, including integrated feature fusion [45], corroborated by Fisher coding and extensive deep networks [62], interactive model of multi-CNN learning [52], and fusing Fisher vectors with CNN data [63].

In [65], the authors built a decision framework using a convolutional neural network to evaluate the PH2 dataset for skin cancer detection. A U-Net could automatically identify malignant tumours, according to [66]. Rodrigues et al. [37] used transfer learning and a CNN as components of their IoT architecture. A hierarchical structure based on two-dimensional elements in the picture and ResNet were presented in [67] for improved deep learning. The following identification techniques were utilized to recognize and estimate essential blood cells in the WBC dataset. A CNN was used to perform classification, as described in [68]. Additionally, Ref. [69] took advantage of a selectivity feature and an SVM. CNNs were offered as a deep learning strategy in [70] for automating the whole operation.

Six well-known classification algorithms were implemented and tested in [87] to validate their performance on the OCT dataset. The algorithms included transfer learning [87] and IFCNN [88]. IFCNN [88] used numerous convolutional features inside of a CNN and a recurrent fusion method to identify OCT images. Huang et al. [89] devised a special layer guided convolutional neural network (LGCNN) to discriminate between the typical retina and three common macular disorders. Kermany et al. [87] introduced an image-based deep learning (IBDL) technique that adjusted the channel’s parameters and was utilized as a feature representation. Sun et al. [90] used sparse coding and dictionary learning based on the scale-invariant feature transform (SIFT) metaphor to identify AMD, DME, and routine images. Ji et al. [91] used Inception V3 via transfer learning as a feature extractor where a CNN was added on top of the pretrained Inception V3 network after eliminating the top layers to detect feature-space alterations.

Overall, our technique allows us to eliminate unnecessary features from high-dimensional representations of the input medical image extracted from a CNN network. Nevertheless, our framework’s primary flaw is that it is time- and memory-intensive. The next stage is to simplify the framework and make it more efficient. Other techniques of augmentation may be investigated in the future to further enhance our current system.

## 6. Conclusions and Future Works

The attractive characteristics of 6G compared to earlier generations of wireless networks have lately generated a considerable attention in business and academic fields. In our study, the developed framework depended on the cloud centre’s classification models being trained before they could be put to work. They were then transmitted to the cloud centre after being analysed at the cloud centre using learned representations from medical images obtained from edge devices on IoT/fog computing nodes. MobileNetV3 was modified and fine-tuned using medical pictures to determine more complex and informative representations and extract image embedding. Furthermore, a novel metaheuristic algorithm that relied on the arithmetic optimization algorithm (AOA) and hunger games search was developed as a feature selection method to filter only relevant features from image embedding. Convergence was accelerated, and feature vectors were improved as a result. In order to determine how well the developed framework model performed, it was sent to a simulated medical imaging cloud centre or assessed using fog computing and a copy of the developed algorithm. The developed framework was tested on the ISIC-2016, PH2, WBC, and OCT datasets. The results showed that the presented technique outperformed other methods already used for feature selection. In addition, the results of the assessments with other new medical image categorization technologies showed that the IoMT technique developed could enhance the overall performance and services. An increased amount of medical information, as well as its use in medical treatment, will be assessed as part of future research. Combining multiple classification techniques is also an intriguing research topic since it may enable practitioners to improve the performance of current approaches. In addition, the hyperparameters optimization of deep learning models can be investigated as using the wrong hyperparameters can limit the performance of the model.

## Figures and Tables

**Table 2:** Classification results (%) of each FS algorithm using two skin datasets (ISIC and PH2). ET is the execution time.

Alg.	Cls.	ISIC						PH2					
		AC	BA	F	R	P	ET	AC	BA	F	R	P	ET
PSO	SVM	85.75	72.04	84.78	85.75	84.68	0.13	96.07	96.73	96.07	96.07	96.10	0.12
	XGB	84.43	73.72	84.14	84.43	83.92	0.26	92.86	93.45	92.88	92.86	93.40	3.61
	KNN	84.17	71.55	83.53	84.17	83.21	0.08	95.71	96.13	95.72	95.71	95.77	0.24
	RF	85.22	74.72	84.90	85.22	84.68	0.33	91.79	91.67	91.85	91.79	92.60	0.56
GWO	SVM	84.43	71.21	83.65	84.43	83.34	0.16	96.07	96.73	96.07	96.07	96.10	0.09
	XGB	82.85	71.73	82.62	82.85	82.43	0.22	92.86	93.75	92.85	92.86	93.43	2.57
	KNN	84.17	72.55	83.73	84.17	83.45	0.06	95.71	96.13	95.72	95.71	95.77	0.17
	RF	85.22	73.71	84.72	85.22	84.46	0.30	91.79	91.67	91.85	91.79	92.60	0.48
MFO	SVM	86.54	73.03	85.57	86.54	85.60	0.15	96.07	96.73	96.07	96.07	96.10	0.09
	XGB	85.49	**76.39**	85.38	85.49	85.28	0.23	93.21	94.05	93.22	93.21	93.58	2.63
	KNN	82.59	71.07	82.31	82.59	82.08	0.06	95.71	96.13	95.72	95.71	95.77	0.17
	RF	85.22	73.71	84.72	85.22	84.46	0.31	92.14	91.96	92.21	92.14	92.87	0.50
ArchOA	SVM	86.28	74.88	85.73	86.28	85.51	0.09	96.07	96.73	96.07	96.07	96.10	0.06
	XGB	83.64	73.23	83.47	83.64	83.32	0.19	91.79	92.56	91.80	91.79	92.60	1.69
	KNN	84.96	74.05	84.59	84.96	84.35	0.05	95.36	95.83	95.37	95.36	95.40	0.11
	RF	85.75	74.55	85.27	85.75	85.03	0.29	93.57	93.75	93.61	93.57	94.00	0.45
OA	SVM	85.49	73.38	84.86	85.49	84.60	0.14	96.07	96.73	96.07	96.07	96.07	0.06
	XGB	84.70	73.39	84.27	84.70	84.01	0.27	93.57	94.35	93.58	93.57	93.97	1.81
	KNN	85.49	74.38	85.04	85.49	84.79	0.07	95.71	96.13	95.72	95.71	95.77	0.12
	RF	86.28	75.88	85.90	86.28	85.69	0.33	92.14	91.96	92.21	92.14	92.87	0.47
BAT	SVM	86.02	72.20	85.00	86.02	84.97	0.10	96.07	96.73	96.07	96.07	96.10	0.08
	XGB	85.75	75.05	85.36	85.75	85.13	0.19	92.86	93.75	92.86	92.86	93.28	2.48
	KNN	82.32	68.39	81.55	82.32	81.12	0.05	95.71	96.13	95.72	95.71	95.77	0.16
	RF	85.75	75.05	85.36	85.75	85.13	0.28	92.50	92.26	92.56	92.50	93.15	0.46
HGS	SVM	84.96	73.05	84.40	84.96	84.12	0.12	96.07	96.73	96.07	96.07	96.07	0.07
	XGB	85.49	75.39	85.22	85.49	85.02	0.23	92.50	93.15	92.52	92.50	93.15	2.07
	KNN	84.96	73.05	84.40	84.96	84.12	0.07	95.71	96.13	95.72	95.71	95.77	0.15
	RF	84.96	74.05	84.59	84.96	84.35	0.30	92.14	92.56	92.18	92.14	92.85	0.47
CGO	SVM	86.02	74.71	85.50	86.02	85.27	0.14	96.07	96.73	96.07	96.07	96.10	0.07
	XGB	84.96	73.05	84.40	84.96	84.12	0.22	93.57	94.35	93.58	93.57	93.97	2.15
	KNN	84.96	73.55	84.50	84.96	84.23	0.06	95.71	96.13	95.72	95.71	95.77	0.14
	RF	85.22	73.21	84.63	85.22	84.36	0.30	92.86	92.56	92.91	92.86	93.44	0.46
AOA	SVM	86.28	74.88	85.73	86.28	85.51	0.16	96.07	96.73	96.07	96.07	96.10	0.11
	XGB	85.75	73.54	85.08	85.75	84.85	0.25	93.93	94.64	93.94	93.93	94.27	2.86
	KNN	84.96	71.04	83.99	84.96	83.78	0.07	95.71	96.13	95.72	95.71	95.77	0.18
	RF	85.49	73.88	84.95	85.49	84.69	0.31	91.43	91.37	91.50	91.43	92.33	0.50
AOAHG	SVM	**87.34**	74.53	**86.47**	**87.34**	**86.53**	0.06	**96.43**	**97.02**	**96.43**	**96.43**	**96.44**	0.10
	XGB	84.43	72.72	83.96	84.43	83.67	0.09	93.57	94.35	93.58	93.57	93.97	3.01
	KNN	84.17	72.55	83.73	84.17	83.45	0.04	95.71	96.13	95.72	95.71	95.77	0.19
	RF	85.75	75.05	85.36	85.75	85.13	0.26	91.79	91.67	91.85	91.79	92.60	0.53

**Table 3:** Classification Results (%) of each FS algorithm using WBC dataset and OCT dataset. ET is the execution time.

Alg.	Cls.	WBC						OCT					
		AC	BA	F	R	P	ET	AC	BA	F	R	P	ET
PSO	SVM	88.46	88.46	88.65	88.46	90.49	1.1	99.28	99.28	99.28	99.28	99.30	16
	XGB	88.42	88.41	88.64	88.42	90.60	58.0	99.17	99.17	99.18	99.17	99.20	178
	KNN	88.42	88.42	88.61	88.42	90.44	8.4	99.28	99.28	99.28	99.28	99.30	3
	RF	88.46	88.46	88.66	88.46	90.53	6.4	99.28	99.28	99.28	99.28	99.30	104
GWO	SVM	88.50	88.50	88.69	88.50	90.51	0.9	99.38	99.38	99.38	99.38	99.40	11
	XGB	88.42	88.42	88.65	88.42	**90.63**	44.3	99.38	99.38	99.38	99.38	99.40	137
	KNN	88.50	88.50	88.69	88.50	90.55	6.5	99.17	99.17	99.18	99.17	99.20	2
	RF	88.42	88.41	88.61	88.42	90.43	5.5	99.38	99.38	99.38	99.38	99.40	90
MFO	SVM	88.54	88.54	88.74	88.54	90.59	0.8	99.38	99.38	99.38	99.38	99.40	9
	XGB	88.50	88.50	88.71	88.50	90.58	42.5	99.38	99.38	99.38	99.38	99.40	119
	KNN	88.50	88.50	88.70	88.50	90.60	6.1	99.17	99.17	99.18	99.17	99.20	2
	RF	88.46	88.46	88.66	88.46	90.55	5.4	99.17	99.17	99.18	99.17	99.20	89
ArchOA	SVM	88.26	88.25	88.44	88.26	90.32	0.4	99.38	99.38	99.38	99.38	99.40	7
	XGB	88.30	88.30	88.52	88.30	90.45	15.7	99.28	99.28	99.28	99.28	99.30	90
	KNN	88.58	88.58	88.75	88.58	90.55	1.9	99.17	99.17	99.18	99.17	99.20	1
	RF	88.46	88.46	88.67	88.46	90.58	3.3	99.17	99.17	99.18	99.17	99.20	74
AO	SVM	88.26	88.25	88.47	88.26	90.44	0.6	99.28	99.28	99.28	99.28	99.30	9
	XGB	88.34	88.33	88.56	88.34	90.48	29.9	99.38	99.38	99.38	99.38	99.40	125
	KNN	88.46	88.46	88.65	88.46	90.51	4.0	99.28	99.28	99.28	99.28	99.30	2
	RF	88.34	88.33	88.55	88.34	90.48	4.4	99.48	99.48	99.48	99.48	99.49	76
BAT	SVM	88.34	88.34	88.51	88.34	90.23	0.8	99.38	99.38	99.38	99.38	99.40	12
	XGB	88.06	88.05	88.31	88.06	90.42	43.0	99.28	99.28	99.28	99.28	99.30	141
	KNN	88.46	88.46	88.63	88.46	90.41	6.5	99.48	99.48	99.48	99.48	99.49	2
	RF	88.30	88.29	88.52	88.30	90.44	5.5	99.28	99.28	99.28	99.28	99.30	88
HGS	SVM	88.54	88.54	88.74	88.54	90.62	0.4	99.59	99.59	99.59	99.59	99.59	9
	XGB	88.26	88.25	88.47	88.26	90.39	20.5	99.38	99.38	99.38	99.38	99.40	122
	KNN	88.38	88.38	88.58	88.38	90.46	2.7	99.17	99.17	99.18	99.17	99.20	2
	RF	88.46	88.46	88.67	88.46	90.62	4.0	99.38	99.38	99.38	99.38	99.40	86
CGO	SVM	88.50	88.50	88.68	88.50	90.49	1.0	99.59	99.59	99.59	99.59	99.59	10
	XGB	87.94	87.93	88.21	87.94	90.41	36.6	99.17	99.17	99.18	99.17	99.20	125
	KNN	88.22	88.21	88.43	88.22	90.32	5.4	99.17	99.17	99.18	99.17	99.20	2
	RF	88.22	88.21	88.44	88.22	90.41	5.2	99.17	99.17	99.18	99.17	99.20	86
AOA	SVM	88.58	88.58	88.76	88.58	90.57	0.8	99.38	99.38	99.38	99.38	99.40	12
	XGB	88.42	88.41	88.62	88.42	90.54	47.6	99.17	99.17	99.18	99.17	99.20	142
	KNN	88.42	88.42	88.61	88.42	90.44	7.4	99.28	99.28	99.28	99.28	99.30	2
	RF	88.42	88.41	88.62	88.42	90.51	5.9	99.07	99.07	99.07	99.07	99.10	86
AOAHG	SVM	**88.62**	**88.62**	**88.80**	**88.62**	90.59	1.0	**99.69**	**99.69**	**99.69**	**99.69**	**99.69**	6
	XGB	88.30	88.30	88.51	88.30	90.40	48.7	99.38	99.38	99.38	99.38	99.40	68
	KNN	88.46	88.46	88.63	88.46	90.42	6.7	99.59	99.59	99.59	99.59	99.59	1
	RF	88.38	88.37	88.59	88.38	90.52	5.8	99.38	99.38	99.38	99.38	99.40	62

**Table 4:** Accuracy (AC) results of the developed method and other existing methods.

DS	Model	AC (%)	Year	Ref.
ISIC	BL-CNN	85.00	2017	[45]
	DCNN-FV	86.81	2018	[62]
	MC-CNN	86.30	2019	[52]
	MFA	86.81	2020	[63]
	AOAHG + SVM	**87.30**	present	Ours
PH2	ANN	92.50	2017	[65]
	DenseNet + SVM	92.00	2020	[66]
	DenseNet + KNN	93.16	2020	[37]
	ResNet + NB	95.40	2021	[67]
	AOAHG + SVM	**96.40**	present	Ours
WBC	CNN + SVM	85.00	2013	[68]
	CNN	87.08	2017	[69]
	CNN + Augm	87.00	2019	[70]
	AOAHG + SVM	**88.60**	present	Ours
OCT	Transfer Learning	80.30	2018	[87]
	IFCNN	87.30	2019	[88]
	LGCNN	89.90	2019	[89]
	IBDL	94.57	2018	[87]
	ScSPM	97.75	2017	[90]
	InceptionV3	98.86	2018	[91]
	AOAHG + SVM	**99.69**	present	Ours

## Data Availability

The data are available from the authors upon request.
